# Development and Validation of a Tool to Assess the Knowledge, Attitude and Perception of Women Towards Screening Mammography

**DOI:** 10.4314/ejhs.v32i6.11

**Published:** 2022-11

**Authors:** Priyanka Chandrasekhar, Winniecia Dkhar, Nitika C Panakkal

**Affiliations:** 1 Department of Medical Imaging Technology, Manipal College of Health Professions, Manipal Academy of Higher Education, Manipal; 2 Department of Medical Imaging Technology, Manipal College of Health Professions, Manipal Academy of Higher Education, Manipal; 3 Department of Medical Imaging Technology, Manipal College of Health Professions, Manipal Academy of Higher Education, Manipal

**Keywords:** Reliability, Screening Mammography, Validity

## Abstract

**Background:**

Breast cancer is a major public health concern in both developing and developed countries. The mortality rate of breast cancer is high in India due to late detection, and lack of knowledge about the screening mammography. The objective of this study was to develop and establish the psychometric properties of the tool.

**Method:**

A qualitative method was used to carry out the development and validation of a tool to assess the knowledge, attitude, and perception of women towards screening mammography using a cross-sectional study design. Data analyzed using the SPSS 16 were descriptive, and Cronbach's alpha coefficient was used to standardize the tool.

**Results:**

The pilot test, content validity, and internal consistency reliability of the final 25 items were found to be highly adequate. Intra- class correlations of this questionnaire were considered acceptable. Cronbach's alpha score was 0.825.

**Conclusion:**

A valid scale has been developed to evaluate the knowledge, attitude, and perception of women toward screening mammography. The scale is adequately responsive to test the knowledge and possesses strong internal consistency. Lack of knowledge among the general population about the screening mammography. It is recommended to conduct an educational program to enhance knowledge about screening mammography. The questionnaires can be used for evaluating the knowledge, attitude, and perception of women toward screening mammography. Broadening the concept for further questionnaires tool development of knowledge, attitude, and perception of women towards screening mammography in other geographical areas.

## Introduction

Breast cancer is the most common cancer in females worldwide. During the year 2018, around 2.1 million women were newly diagnosed with breast cancer, i.e. about one in 4 of all new cancer cases among women, and the cancer is the most common in 154 of the 185 countries included in GLOBOCAN (1). Breast cancer is the fifth leading cancer of death (627,000 deaths, 6.6%) because the prognosis is good in developed countries. Inadequate knowledge of the disease and limited awareness of the risk factors, fear of disease, and different social and psychological reasons are the main causes for ignoring and hiding the disease and by the time the patients report to the hospital, the disease is in its late stages.

Early detection of breast cancer and early presentation for management has been shown to reduce mortality rates and it is therefore important that regular screening methods be encouraged among the population. Mammography is used in both women's having no symptoms and experiencing symptoms. It is a screening tool to detect early breast cancer (2). It is classified into diagnostic and screening mammography. Diagnostic mammography is used if a patient has signs and symptoms related to breast cancer to find the abnormalities and for follow-up studies. Screening mammography is performed in women with no signs and symptoms of breast cancer if any abnormalities there we can identify them as early as possible. Breast cancer screening was done according to age, medical and family history. The driving force behind the widespread institution of screening mammography is studies showing that screening mammography results in a substantial decrease in breast cancer mortality by approximately 30% (3,4). Developing countries like India are lagging in proper utilization of screening tools in the health care system (5,6). In most females, there is a lack of awareness regarding breast cancer symptoms and risk factors showing very less breast cancer screening practices. Many studies have been done to increase women's awareness and screening tools to reduce breast cancer risk factors (7,8).

In India, we still lack such guidelines, which need to be formulated at the earliest since the percentage of breast cancer cases and the death rate is increasing with each passing year which may be due to a lack of awareness of the disease or screening tool. With respect to the above-described scenario, the present study is intended to develop and test a screening tool for knowledge, attitude, and perception of screening mammography in carcinoma of the breast.

In India there is a lack of standardized instruments to assess the woman's knowledge, awareness, and practice of Mammography, therefore the main objective of this study was to develop and establish the psychometric properties of the tool. The screening tool is one of the simplest ways to create awareness regarding breast cancer and mammography, in order to curb the occurrence rate and also mortality rate in India.

## Methods

This cross-sectional study was conducted over a period of 15 months. This study population was consisting of 15 subjects for phase I and 60 subjects for phase II between 20 to 60 years of females with a minimum qualification of 12th standard graduation. The socio-demographic data are listed in Table 1.

**Table 1:** Socio-demographic data of the study participants (n=60)

Characteristics	N (%)
***Age (years)***	
20–30	45(75%)
31–40	11(18.3%)
41–60	04(7.0%)
***Education***	
12^th^ Qualified	11(18%)
Under graduation	26(43%)
Post-Graduation	23(38%)
***Occupation***	
Health profession	09(15%)
Academics	09(15%)
Others	43(71.6%)
***Origin***	
Urban	28(46.6%)
Rural	32(53%)

### Phase I: Qualitative Research

Phase I of the study was qualitative research, which was intended to access knowledge, attitude, and perception toward screening mammography. The planning phase involved a detailed review of the literature to determine the concept and construct the awareness. After an in-depth interview with 15 individuals and theoretical saturation was reached, deficient areas of awareness had been identified and the questionnaire/tool was constructed and items were generated.

### Phase II: Validation

In phase II three subjects in the field of medical imaging and mammography were validated and the items were generated from phase I under three categories: subject content, applicability to the Indian context, and redundancy. The validated questionnaires/items underwent phase III.

### Phase III: Reliability

In phase III Retest reliability of the scale was established. The test was re-administered after 3 weeks with the same sample size to test the reliability of the scale. Intra-class correlation of 0.60 was considered marginal, a value of 0.70 was considered acceptable and a correlation of 0.80 was considered as high. Cronbach's alpha score was used to measure the internal consistency and a score of 0.70 was considered an acceptable value for scale reliability.

## Results

### Phase I: Qualitative Study

A total of fifteen subjects were interviewed for this study. All subject's interviews were carried out in English. All the interviews were audio recorded. The analysis of the qualitative study explained that many of the participants had very poor knowledge of the basic anatomy and physiology of breasts. Two third women said that breast cancer occurred due to tumors or lumps in the breast. Many participants told that breast cancer can occur due to family history. Remaining said that breast cancer could be due to dietary habits, smoking, stress, consumption of alcohol, and environmental factors like radiation and chemicals. Most of the participants lacked knowledge about the risk factors, signs, and symptoms of breast cancer.

When participants were asked about screening tools for breast cancer, it was noted that breast self-examination and mammography were used to detect the primary stage of cancer. More than 50% percent of the women were not practicing self-breast examination. Although many participants identified as a preventive examination, their responses regarding how often mammography should be done were varied. Approximately 50% of participants said that women should undergo mammography yearly after attaining 40 years and the remaining participants reported that they are not aware of how often mammography should be done. Most of the participants said that they would undergo mammography only if referred by a physician. After a phase I, the results showed that there were deficient areas of awareness and the questionnaire/tool was constructed and item was generated accordingly.

### Phase II: Validity

After grouping, the items were subjected to content validation by experts in the field of medical imaging. A total of twenty-five questions were generated among which twenty-two questions were included in the questionnaire based on their understanding, relevance, and appropriateness. Three questions were altered by the subject experts because they were biased.

### Phase III: Reliability

The reliability of the instrument was assessed by using a standardized questionnaire. Questionnaires were given to women who were included in this study. The test was re-administered after 3 weeks with the same sample size to test the reliability of the scale. Results of this test-retest reliability of the women toward screening mammography were shown in Tables 2, and 3. Intra-class correlations of this questionnaire were considered acceptable. Items that have less than 60% agreement were considered less acceptable because most of the women responded “no” in both test and retest. The percentage agreement between the two items for each question was acceptable.

**Table 2:** Test-retest reliability of the women towards screening mammography

Knowledge of Breast Anatomy & Physiology	Percentage of Agreement
Age breast starts to develop	76.6%
Composition of breast tissue	65%
Menopause starts	66.6%
Menarche starts	80%

**Table 3:** Test-retest reliability of the women towards screening mammography

Knowledge on Breast Cancer	No. (%)
Breast cancer knowledge	80%
**Knowledge about signs and symptoms**	
Lump in the breast	75%
Pain or soreness of the breast	73%
Change in the size of the breast	71%
Discoloration and dimpling of the breast	68.3%
Lump under the arm pit	66.6%
**Risk factors for the breast cancer**	
Age	60%
Family history	66.6%
High fat diet	60%
Smoking tobacco	66.6%
Alcohol consumption	56.6%
First child at late age	60%
Early onset of menarche	61.6%
Menopause	66.6%
Radiation workers	65%
Fertility drugs	60%
**Screening tool for breast cancer**	
Heard of Breast self -examination	80%
Practicing Breast self -examination	70%
How often should you practice BSE	75%
Methods you know that help in detecting breast cancer	68.3%
Know about screening mammography	70%
At what age should you go for an mammography	68.3%
How often screening mammography should be done	60%
Is mammography a useful tool for early detection of Breast Cancer	68.3%
**Pros and cons of mammography**	
Mammography is a painful procedure?	63.3%
Mammography is a costly investigation?	60%
Mammography involves in radiation?	68.3%
All females have an equal chance of getting BC?	55%
Mammography is needed for females with breast abnormalities?	73.3%
Will you go for mammography only on your physician's advice?	70%

**Internal consistency reliability**: Cronbach's alpha score was used to measure the internal consistency of items. Total items showed good internal consistency (0.825).

## Discussion

In light of the increasing risk of breast cancer worldwide, understanding women's attitudes and perceptions regarding screening mammography is essential. Primary screening of women for breast cancer is the first step toward preventing the disease. Developed countries have recognized these many years back. With limited resources available in developing countries it's difficult to access breast cancer affecting women.

Our study aimed to develop and validate a tool to access the knowledge, attitude, and perception of women towards screening mammography. Most of the previous studies were done to estimate the knowledge, attitude, and perception toward the screening mammography and breast cancer (7–12), however, in the present study, we developed and validated the standardized questionnaires to assess the knowledge, attitude, and perception of women towards screening mammography. The study utilized closed-ended questionnaires comprising mainly demographic data, basic knowledge of breast anatomy and physiology, knowledge about breast cancer, a screening tool for breast cancer, and the pros and cons of mammography. Other studies also mainly focused on demographic data, knowledge about breast cancer, and screening practices (9,10).

Demographic data included age, level of education, and occupation of the participants. Questions about their knowledge, attitudes, and practices regarding breast cancer were also asked. In the present study, all the participants were employed irrespective of their educational qualifications. Working status determines participants' interest in healthcare needs by checking other factors we can assess the acceptance of medical services. The tool used for this study was a self-administrated, pre-tested, structured questionnaire validated by the experts. Whereas other similar studies have used either a standard questionnaire or conducted a structured interview for their studies. The study populations in the previous studies were women from rural and urban areas and one study done in morocco was conducted on general practitioners (9).

This study primarily focuses on the general population in the rural and urban areas in the south coastal region. Phase I of our study was a qualitative analysis of data where a total of fifteen subjects were interviewed and recorded by audio tape to ensure a more data collecting process. In this study, most of the participants have less information about the risk factors, signs, and symptoms of breast cancer. Most of the participants identified breast self-examination and mammography as used for primary diagnosis of breast cancer. More than 50% of the women are not practicing self-breast examinations. Results showed that participants have less knowledge about breast cancer and screening methods. These results corresponded with a similar study done by Nitin Gangane et al (11) in a rural area in central India. They found that less than 50% of rural and urban women heard about breast cancer and screening mammography. However, the knowledge about breast cancer was equally poor in both rural and urban women. Urban women showed more interest in breast cancer screening practices. In this study test-retest, the reliability of the scale was moderate and the percentage of agreements was good for many questions. We found that participants lacked information about the signs and symptoms and risk factors of breast cancer.

The validated questionnaire was suitable for surveys of knowledge, attitude, and perception of screening mammography of women. A similar study conducted by Louise Linsell et al (11) in London explained that the reliability of the Breast Cancer Awareness Measures (BCAM) of many items was good and reported that on-medical persons had less knowledge compared to the medical persons. The questionnaire showed good internal consistency and validity. In the present study, Cronbach's alpha score was used to measure the internal consistency of items. Total items having good internal consistency, Cronbach's alpha value was 0.825. Other similar studies also used a Cronbach alpha value was >0.70 for all three dependent variables and internal consistency was high.

We recommend further study to conduct an educational program to enhance knowledge about screening mammography and these questionnaires can be used for evaluating the knowledge, attitude, and perception of women towards screening mammography. Broadening the concept for further questionnaires tool development of knowledge, attitude, and perception of women towards screening mammography in other geographical areas should also be done.

Therefore, a valid scale had been developed to evaluate the knowledge, attitude, and perception of women toward screening mammography. The scale was adequately responsive to test the knowledge and possess strong internal consistency and we concluded that there was a lack of knowledge among the general population about the screening mammography, which may be one of the prime reasons for the higher mortality rate due to breast cancer in India.
